# Association of Folic Acid Supplementation in Early Pregnancy with Risk of Gestational Diabetes Mellitus: A Longitudinal Study

**DOI:** 10.3390/nu14194061

**Published:** 2022-09-29

**Authors:** Hongyan Chen, Yaqiong Hu, Yannan Li, Wenzheng Zhou, Niya Zhou, Huan Yang, Qing Chen, Yawen Li, Qiao Huang, Zhen Chen

**Affiliations:** 1 Department of Quality Management, Chongqing Health Center for Women and Children, Women and Children’s Hospital of Chongqing Medical University, Chongqing 401147, China; 2 Clinical Research Center, Chongqing Health Center for Women and Children, Women and Children’s Hospital of Chongqing Medical University, Chongqing 401147, China; 3 Key Lab of Medical Protection for Electromagnetic Radiation, Ministry of Education of China, Institute of Toxicology, College of Preventive Medicine, Third Military Medical University, Army Medical University, Chongqing 400038, China; 4 Department of Obstetrics and Gynecology, Chongqing Health Center for Women and Children, Women and Children’s Hospital of Chongqing Medical University, Chongqing 401147, China

**Keywords:** folic acid, gestational diabetes mellitus, dose, duration, risk

## Abstract

Background: Gestational diabetes mellitus (GDM) may lead to many adverse effects on women and their offspring. Method: 24,429 pregnant women were enrolled during early pregnancy from January 2018 to December 2021. The self-reported intake of folic acid supplements was assessed via a questionnaire. Oral glucose tolerance tests were used for the diagnosis of GDM. The association between intake or not, dose, and duration of folic acid and GDM risk was assessed. Results: 6396 (26.18%) women were diagnosed with GDM. In the univariate models, folic acid was found to be correlated with total GDM risk (OR = 0.82, 95% CI: 0.70~0.95, *p* = 0.009). After adjusting for potential confounders, the association with total GDM risk was not significant, but the association of folic acid with 2-h PBG diagnosed GDM risk was consistently significant (OR = 0.75, 95% CI: 0.63~0.90, *p* = 0.002). No significant association between the dose and duration of folic acid supplementation and GDM risk was observed in the analyses. Conclusion: Folic acid supplementation might be a protective factor for the risk of GDM caused by the high level of postprandial blood glucose, but the dose or duration-related association between folic acid supplementation and GDM risk is not clear.

## 1. Introduction

Gestational diabetes mellitus (GDM) refers to impaired glucose tolerance or diabetes that first occurs during pregnancy. As one of the countries with the fastest-growing incidence of diabetes in the world, China has a high GDM prevalence of 14.8% (95% CI: 12.8–16.7%) according to the latest systematic review and meta-analysis including 25 cross-sectional studies or retrospective studies [1]. Studies have shown that GDM may lead to type 2 diabetes mellitus (T2DM), gestational hypertension, and preeclampsia of mothers, macrosomia, premature birth, neonatal hypoglycemia, jaundice, respiratory distress, and pneumonia of the offspring, it is also related to childhood obesity and increased risk of cardiovascular disease in adulthood [1,2,3,4,5].

Folate is a B vitamin that naturally exists in vegetables, and fruits, and which cannot be synthesized by humans. During food storage or cooking, folate is partially lost. Folic acid, the synthetic form of folate, is mainly obtained from dietary supplements and fortified foods and has higher bioavailability than folate [6]. Due to its role in DNA methylation and biosynthesis of nucleic acids and proteins, the demand for folic acid is higher during pregnancy to support cellular replication and fetal growth [7,8,9]. Studies have shown a lack of folic acid during pregnancy may lead to habitual abortion, premature birth, fetal dyspepsia, and growth retardation [10,11,12]. Folic acid supplementation is also recommended by many countries for preventing neural tube defects (NTD) before and during pregnancy [13,14,15]. After a two-year monitoring program among pregnant women in areas with high incidence of NTD, China launched a folic acid supplement project nationwide in 2009, suggesting a 400 μg/day of folic acid supplementation three months before pregnancy and during early pregnancy [16].

Some studies have suggested that folic acid supplementation is associated with the development of GDM [17,18,19,20,21], but the conclusions are equivocal. A previous study [19] included 14,553 women in the Nurses’ Health Study II cohort and showed that folic acid supplementation before pregnancy was negatively correlated with the risk of GDM. They also found that folic acid in supplements had higher biological effects than folate in food. However, the China–Anhui Birth Cohort Study with 1938 pregnancies found that daily folic acid supplementation in early pregnancy increased the risk of GDM, with an OR of 2.25 (95% CI: 1.35~3.76) [22]. Another cohort from Wuhan Tongji [20] found that 800 μg/day or more folic acid supplementation from pre-pregnancy through mid-pregnancy was associated with a higher GDM risk (OR = 2.09, 95% CI: 1.30~3.36), but the association was not observed in other dose groups. In view of the different diabetes susceptibility levels and study periods in previous research, this study aimed to explore the relationship between folic acid supplementation during early pregnancy and the risk of GDM in a large-scale longitudinal study in Chinese women. In a subgroup of the study sample, we also examined whether the association was related to the dosage and duration of folic acid supplementation.

## 2. Materials and Methods

### 2.1. Study Population

**Figure 1:**
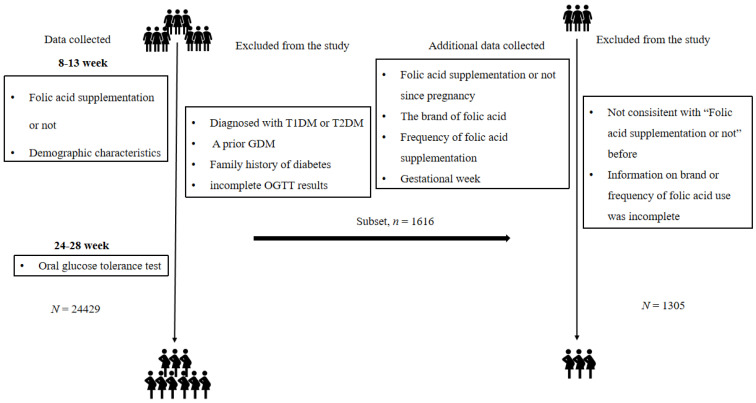
Study flowchart. T1DM, type 1 diabetes mellitus; T2DM, type 2 diabetes mellitus; GDM, gestational diabetes mellitus; OGTT, oral glucose tolerance test.

A total of 24,429 pregnant women who attended Chongqing Health Center for Women and Children, China, from January 2018 to December 2021 were recruited. The recruitment criteria were as follows: Chinese women aged 18–45 years old; 8–13 weeks of gestation; registered the information of “Folic acid supplementation or not”. Exclusion criteria: diagnosed with T1DM or T2DM or prior GDM; had a family history of diabetes; incomplete oral glucose tolerance test (OGTT) results. A subset of participants (*n* = 1305) volunteered to complete a more detailed self-report folic acid questionnaire, including four questions: (1) Have you taken folic acid during this pregnancy; (2) What brand of folic acid supplements are you taking; (3) The frequency of the folic acid supplementation per week; (4) Gestational week (Figure 1).

### 2.2. Measurement of Folic Acid Supplementation, Blood Glucose, and Diagnosis of GDM

The folic acid dose per tablet was determined by the brand and corresponding ingredient list. The average daily folic acid intake from supplements was calculated based on the frequency of folic acid supplements per week. A 75-g OGTT during 24–28 weeks gestation was used to measure the blood glucose concentration of the participants. The details of the OGTT method are described elsewhere [23]. The diagnosis of GDM was made if one or more of the following glucose values were met: fasting blood glucose (FBG) ≥ 5.1 mmol/L, or 1-h post blood glucose (1-h PBG) ≥ 10.0 mmol/L, or 2-h post blood glucose (2-h PBG) ≥ 8.5 mmol/L [24].

### 2.3. Covariates

Women reported their age, height, weight before pregnancy, gravidity, parity, smoking history, drinking history, singleton/multiple pregnancy, and the macrosomia history. Pre-pregnancy body mass index (BMI) was calculated using height and the weight before pregnancy. Then, pre-pregnancy BMI was categorized according to the Chinese criteria: underweight (BMI < 18.5 kg/m^2^), normal weight (18.5 kg/m^2^ ≤ BMI < 24.0 kg/m^2^), overweight (24.0 kg/m^2^ ≤ BMI < 28.0 kg/m^2^), and obese (BMI ≥ 28.0 kg/m^2^) [25].

### 2.4. Statistical Analysis

Differences across the categories were compared using a Chi-square test for categorical variables and *t*-test for continuous variables. The Shapiro–Wilk test and histogram were performed to assess the normality of data. Logistic regression models were used to estimate the odds ratios (ORs) and 95% CIs (confidence intervals) of GDM risk (total, FBG diagnosed, 1-h PBG diagnosed, and 2-h PBG diagnosed) associated with folic acid supplementation or not in 24,429 pregnant women. Furthermore, logistic regression models were used to assess the association between three categories of folic acid supplementation (dose: <400, 400–800, and ≥800 μg/day; duration: <3 months and ≥3 months; dose and duration, respectively) and the risk of GDM in 1305 women with the detailed folic acid questionnaire. Age, pre-pregnancy BMI, gravidity, parity, smoking history, drinking history, number of pregnancies, the macrosomia history, and gestational week were included as confounders in all models. Two tailed *p* values < 0.05 were deemed to represent statistical significance. All statistical evaluations were performed with IBM SPSS version 25.0.

## 3. Results

### 3.1. Demographic Characteristics of the Participants

Among the 24,429 women recruited in the first trimester, the average age was 32.35 ± 4.69 years, and the average pre-pregnancy BMI was 21.44 ± 3.50 kg/m^2^. Of all the pregnant women, 96.63% reported regularly folic acid intake while 3.37% (*n* = 823) did not. The average FBG, 1-h PBG, and 2-h PBG were 4.48 ± 0.43 mmol/L, 8.04 ± 1.89 mmol/L, 6.90 ± 1.57 mmol/L, respectively. A total of 0.11% (*n* = 28) had a history of macrosomia. A total of 26.18% (*n* = 6396) women were diagnosed with GDM later in the second trimester. Of all GDM women, 2154 (33.65%) were diagnosed with FBG (≥5.1 mmol/L), 3920 (61.24%) were diagnosed with 1-h PBG (≥10.0 mmol/L), and 3857 (60.25%) were diagnosed with 2-h PBG (≥8.5 mmol/L). Furthermore, 2857 (11.70%) women had a blood glucose level exceeding the standard at two time points, with 777 (3.18%) women at three time points. When comparing the GDM group to the non-GDM group, a higher proportion of advanced age (33.27 ± 4.73 years vs. 32.02 ± 4.63 years, *p* < 0.001), greater BMI (22.50 ± 3.33 kg/m^2^ vs. 21.06 ± 3.49 kg/m^2^, *p* < 0.001), and lower folic acid supplement rate (96.12% vs. 96.81%, *p* = 0.009) were observed in the GDM group (Table 1).

Among the 1305 women with a completed folic acid information questionnaire, the average age was 30.14 ± 4.01 years, and the average pre-pregnancy BMI was 21.59 ± 3.18 kg/m^2^. Of the pregnant women, 99.54% reported regular folic acid intake while only 0.46% (*n* = 6) did not. The average FBG, 1-h PBG, and 2-h PBG were 4.50 ± 0.45 mmol/L, 8.20 ± 1.80 mmol/L, and 7.03 ± 1.53 mmol/L, respectively. A total of 26.89% (*n* = 351) women were diagnosed with GDM. When comparing the GDM group to the non-GDM group, a higher proportion of advanced age (30.73 ± 3.74 years vs. 29.92 ± 4.08 years, *p* = 0.001) and greater BMI (22.41 ± 3.15 kg/m^2^ vs. 21.28 ± 3.14 kg/m^2^, *p* < 0.001) were observed in the GDM group, but this was not found in the folic acid supplement group (99.43% vs. 99.58%, *p* = 0.663) (Table S1).

### 3.2. Folic Acid Supplementation and the Risk of GDM

Before the multivariate analysis, the association between folic acid supplementation and age, pre-pregnancy BMI, the number of pregnancies, and macrosomia history was explored, and it was found that age (χ^2^ = 41.20, *p* < 0.001) and pre-pregnancy BMI (χ^2^ = 10.25, *p* = 0.017) were significantly associated with folic acid supplementation. A higher proportion of women of a younger age (<25 years, 4.50% vs. 2.35%), advanced age (≥35 years, 35.97% vs. 30.54%), overweight (25 kg/m^2^ ≤ BMI < 30 kg/m^2^, 15.07% vs. 12.97%), and with obesity (BMI ≥ 30 kg/m^2^, 2.92% vs. 1.83%) were found more likely not to take folic acid supplements. There was no difference in the numbers of multiple pregnancies or women with a history of macrosomia between the groups of folic acid supplementation or not.

Table 2 shows the logistic regression results of the association between folic acid supplementation and total risk of GDM, 1-h PBG diagnosed GDM, and 2-h PBG diagnosed GDM, respectively. In the univariate models, folic acid supplementation was found to be correlated with total GDM risk (OR = 0.82, 95% CI: 0.70~0.95, *p* = 0.009), 1-h PBG diagnosed GDM risk (OR = 0.80, 95% CI: 0.67~0.95, *p* = 0.012), and 2-h PBG diagnosed GDM (OR = 0.71, 95% CI: 0.60~0.85, *p* < 0.001), respectively. When the age, pre-pregnancy BMI, gravidity, parity, smoking history, drinking history, and macrosomia history were adjusted for, the association with total GDM risk disappeared. However, the association with 2-h PBG diagnosed GDM was still significant and generally unchanged (OR = 0.75, 95% CI: 0.63~0.90, *p* = 0.002). Furthermore, when the adjustment for age and BMI was excluded, other potential confounders had little effect on the results.

### 3.3. Dose and Duration of Folic Acid Supplementation and the Risk of GDM

A total of 1305 pregnant women with detailed information of folic acid usage were categorized into the groups below based on the doses (<400, 400–800, and ≥800 μg/day) and durations (<3, and ≥3 months) of supplementation. Meanwhile, gestational week was also included in the potential confounders adjustment process in the multivariate logistic regression models. For the folic acid supplementation of 1305 pregnant women, 73.56% with a dose ≥800 μg/day, 85.66% for less than 3 months, and 64.01% both took folic acid with a dose ≥800 μg/day and a duration of less than 3 months. Since the questionnaire of folic acid supplementation was finished during 8–13 weeks of pregnancy, and the average daily intake of most recruited women was ≥800 μg/day, the last group was listed as the reference group. No significant association between the dose and duration of folic acid and GDM risk was observed in the analyses (Table 3).

### 3.4. Sensitivity Analyses

**Figure 2:**
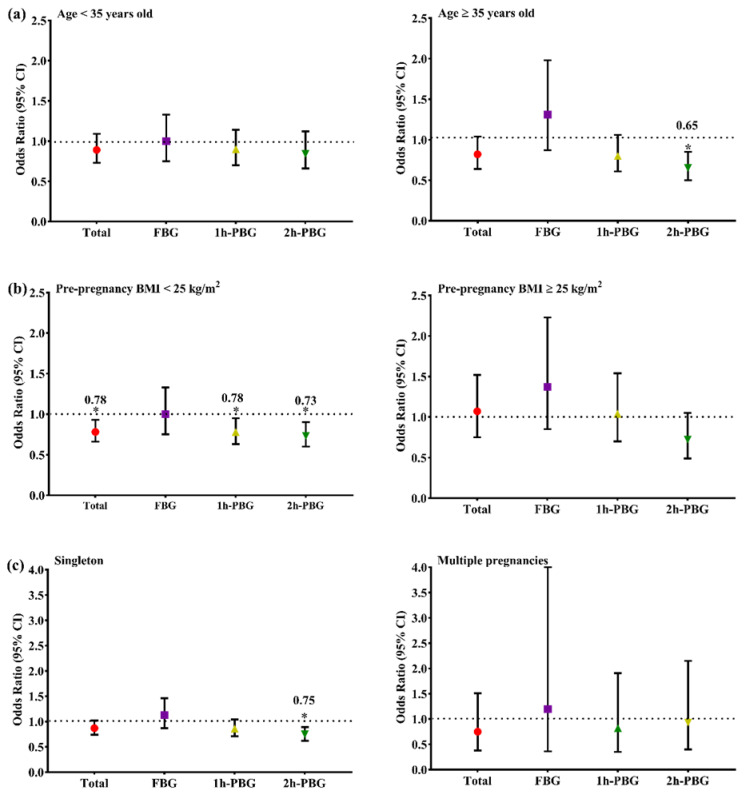
ORs and 95% CIs for GDM associated with folic acid supplementation in subgroups, stratified by age (**a**), pre-pregnancy BMI (**b**), number of pregnancies (**c**). All groups were adjusted for age, pre-pregnancy BMI, gravidity, parity, smoking history, drinking history, number of pregnancies, and macrosomia history. * *p* < 0.05.

The participants were divided into six different subgroups by age (<35 years, 16,923 (69.27%) versus ≥35 years, 7506 (30.73%)), pre-pregnancy BMI (<25 kg/m^2^, 20788 (85.10%) versus ≥25 kg/m^2^, 3641 (14.90%)), and the number of pregnancies (singleton, 22,897 (93.73%) versus multiple pregnancies, 1532 (6.27%)) to analyze the association between folic acid supplementation and the risk of GDM. The results are shown in Figure 2. The inversely significant association between folic acid supplementation and total GDM risk was only observed in the group of pre-pregnancy BMI < 25 kg/m^2^ (OR = 0.78, 95% CI: 0.66~0.93, *p* = 0.006). For the FBG diagnosed GDM women, no significance was observed in any subgroups. For the 1-h PBG diagnosed GDM women, only the group of pre-pregnancy BMI < 25 kg/m^2^ (OR = 0.78, 95% CI: 0.63~0.95, *p* = 0.016) was found to be significant for the association between folic acid supplementation and GDM risk. For the association between the risk of 2-h PBG diagnosed GDM and folic acid supplement, there were significant associations for the group of age ≥ 35 years (OR = 0.65, 95% CI: 0.50~0.85, *p* = 0.001), BMI < 25 kg/m^2^ (OR = 0.73, 95% CI: 0.60~0.90, *p* = 0.002), and singleton pregnancy (OR = 0.75, 95% CI: 0.62~0.89, *p* = 0.001) (Table A1).

## 4. Discussion

In this study, the association between folic acid supplementation in early pregnancy and GDM was explored based on a large-scale group of Chinese women. Folic acid supplementation might be a protective factor against the risk of GDM caused by a high level of postprandial blood glucose, especially in women with normal pre-pregnancy BMI. However, dose or duration-related association between folic acid supplementation and the risk of GDM was not observed, which was inconsistent with previous studies.

Currently, several methods are used to evaluate folic status, and each has its advantages. Serum folic acid indicates the short-term folic acid status, and rapidly responds to folic acid supplementation, while red blood cell folic acid indicates the long-term (nearly 3 months) status. Both methods are able to accurately evaluate the status of folic acid in vivo [18]. However, as a simple and relative accurate method, a food frequency questionnaire is also widely used to assess the relationship between nutrient intake and risk of disease. A study [19] based on the Nurses’ Health Study II used a food frequency questionnaire to assess folate intake. They not only took supplemental folic acid into consideration, but also food folate intake and total folate intake. After adjusting for intake of multivitamins and other micronutrients, women with 1–399, 400–599, and ≥600 μg/day of supplemental folic acid intake before pregnancy had RRs of GDM of 0.83, 0.77, and 0.70, respectively (*p* = 0.002). The results that folic acid supplementation is a protective factor for GDM is partly consistent with this study. However, the dose-related association finding was inconsistent with our research. Furthermore, the GDM diagnosis of their study was self-reported on the questionnaire based on their National Diabetes Data Group criteria, which was different to our study. The China–Anhui Birth Cohort Study C-ABCS [22], which included 1938 pregnant women, suggested that folic acid supplementation in early pregnancy increases the risk of GDM (OR = 2.25, 95% CI: 1.35~3.76). Especially in women with a pre-pregnancy BMI ≥ 25 kg/m^2^, the risk of GDM was much higher (OR = 5.63, 95% CI: 2.77~11.46). In the sensitivity analyses of our study, an inverse protective association between folic acid supplementation and the risk of GDM was also absent in women with a pre-pregnancy BMI ≥ 25 kg/m^2^. We do not know whether other covariates except for pre-pregnancy BMI and family history were adjusted for, as the article of C-ABCS did not specify. In the present study, women with a family history were excluded. A Danish cross-sectional study [26] showed no clear association between folic acid use and the risk of GDM. However, the diagnosis of GDM was from a self-report by the pregnant women and only 62.1% of women used folic acid supplements or a multivitamin, which was lower than that of our study participants. Two cohort studies [20,25] in China showed that both longer duration and higher doses had a positive correlation with the risk of GDM. We did not find the same results for women with folic acid supplementation when the dose and duration were taken into account in the first trimester. One of them [25], which included 950 mother–offspring pairs, found that folic acid supplementation for ≥3 months before pregnancy was correlated with an increased risk of GDM (aRR = 1.72, 95% CI: 1.17~2.53). Comparatively, the sample size of this study was relatively small and the study period was different. Another study with 4353 participants [20] showed that peri-conception folic acid supplement use (≥800 μg/day) increased the later risk of GDM, but no significant association was observed in other groups. The study period and time of recruitment were both longer than that of our study. In addition, both of the studies excluded women with multiple pregnancies. In the above studies, except for two [19,26], GDM was confirmed with the 75-g OGTT diagnostic criteria, which was the same as this study.

In our study, the folic acid supplementation rate was 96.63%, and the prevalence of GDM in our study was 26.20%, which was higher than previous studies [18,20,22,25]. The hospital where this study was conducted is the only municipal health center for women and children; therefore, pregnant women or patients are more likely to come to this hospital. Multiple pregnancies and a history of macrosomia are two recognized risk factors of GDM. The rate of multiple pregnancies in this study was 6.27% and only 29 women had a history of macrosomia. We also analyzed the association between folic acid supplementation and other possible risk factors and found that age and pre-pregnancy BMI were both significantly associated with folic acid supplementation and the risk of GDM. In the univariate analysis of folic acid supplementation and GDM, a significant inverse association was observed. After adjusting for the potential confounders excluding age and pre-pregnancy BMI, the significance of the association hardly changed. However, after adjusting for all the potential confounders this study, the significant association disappeared. Therefore, covariates other than age and pre-pregnancy BMI had little effect on the association. In the sensitivity analyses, the association between folic acid supplementation and the risk of GDM was mainly found in women of normal pre-pregnancy BMI. These results also support that controlling weight gain is still the main preventive measure for GDM [18,27].

In the present study, the main significant results were observed in the association between folic acid supplementation and 2-h PBG diagnosed GDM. In a previous study about sleep quality and the risk of GDM, we also found that the Pittsburgh Sleep Quality Index was significantly associated with blood glucose level, especially for postprandial blood glucose [23]. A study [28] based on the National Health and Nutrition Examination Survey (NHANES) from 2005 to 2016 found that the prevalence of diabetes was underestimated without considering 2-h PBG even though OGTT was time-consuming and burdensome. Furthermore, a high result on the 2-h PBG was also associated with an increased risk of cardiovascular disease and all-cause mortality [29]. Therefore, 2-h PBG might be considered as a sensitive indicator for diagnosing diabetes and predicting the association between exposure factors and diabetes. Associations that could not be seen at other stages showed up in the 2-h PBG analyses.

The possible mechanisms may include insulin resistance, which is considered as contributing to the etiology of GDM [30]. Animal experiments studies [31,32] showed that inadequate folate intake might have an effect on insulin resistance though the compromised methylation capacity and impaired phosphatidylcholine synthesis. Moreover, folic acid supplementation may upregulate AMPK to improve insulin resistance of mice fed with a high-fat diet. Another mechanism may be the protective effect against oxidative stress directly or through lowering blood homocysteine levels indirectly. High homocysteine has been associated with insulin resistance. Some studies have speculated that a sufficient pool of methyl donors may lower homocysteine levels leading to reduced oxidative stress and systemic inflammation, both of which can interfere the insulin effect [32,33,34,35,36]. One previous study has also suggested that folic acid supplementation can increase the wound healing rate and reduce the healing time required via suppressing oxidative stress [37].

### Strengths and Limitations

The main advantages of this study are derived from the large sample-sized longitudinal study design for providing adequate statistical power and a prospective view. Moreover, the diagnosis of GDM was totally based on the 75-g OGTT instead of self-reported results. The study also has some limitations. Estimates of folate status in women of childbearing age in Southwest China are lacking. However, according to previous studies [38,39], the overall folate status in South China is higher than before, but still lower than the WHO RBC folate concentration threshold of 906 nmol/L for NTD prevention [40]. The dietary structure of residents in Southwest China is complex, but the overall dietary habits are relatively consistent. Therefore, the basal intake of folate is relatively consistent. A previous study showed that folic acid supplements were almost the only source of folic acid besides dietary intake, and folic acid in supplements is more bioavailable than that of food [41]. Even though the information of dietary folic acid intake and different eating habits between urban and rural areas was not recorded and the potential confounders of diet were not examined, the assessments of folic acid intake were relatively accurate. Some studies have suggested that an imbalance between vitamin B12 and folate was associated with GDM risk [42,43,44], but we did not collect data on vitamin B12. The method of folic acid supplementation for most pregnant women in this study was using a multivitamin. Therefore, it was difficult to consider the effect of other nutrients separately, hence there might be other possible confounders that have not been taken into account here. The measurement of serum or RBC folate level might be needed in combination with the questionnaire study in the same population in further studies.

## 5. Conclusions

The present study found that the association of folic acid supplementation with GDM was ambiguous, but it might be a protective factor against the risk of GDM caused by a high level of postprandial blood glucose, especially in women with normal pre-pregnancy BMI. The dose or duration-related association between folic acid supplementation and the risk of GDM was not obvious, and more studies are needed to explore this open question.

## Figures and Tables

**Table 1:** Demographic characteristics of the study population.

Characteristics		Total	GDM	Non-GDM	*χ*^2^/*t*	*p*
			N (%)	N (%)		
Age (years)	Mean ± SD	32.35 ± 4.69	33.27 ± 4.73	32.02 ± 4.63	18.45	<0.001
	<25	592 (2.42)	81 (1.27)	511 (2.83)	333.94	<0.001
	25~30	5964 (24.41)	1152 (18.01)	4812 (26.68)		
	30~35	10,367 (42.44)	2747 (42.95)	7620 (42.26)		
	≥35	7506 (30.73)	2416 (37.77)	5090 (28.23)		
Pre-pregnancy BMI (kg/m^2^)	Mean ± SD	21.44 ± 3.50	22.50 ± 3.33	21.06 ± 3.49	28.72	<0.001
	<18.5	5648 (23.12)	605 (9.46)	5043 (27.97)	1024.49	<0.001
	18.5~23.9	13,689 (56.04)	3934 (61.51)	9755 (54.10)		
	24.0~27.9	4147 (16.98)	1512 (23.64)	2635 (14.61)		
	≥28	945 (3.87)	345 (5.39)	600 (3.33)		
Gravidity	1	8072 (33.05)	2135 (33.39)	5937 (32.93)	0.92	0.631
	2	6668 (27.30)	1718 (26.86)	4950 (27.46)		
	≥3	9684 (39.65)	2542 (39.75)	7142 (39.61)		
Parity	0	15,332 (62.77)	4056 (63.42)	11,276 (62.54)	1.58	0.209
	≥1	9093 (37.32)	2339 (36.58)	6754 (37.46)		
Smoking	Yes	696 (2.90)	199 (3.19)	497 (2.80)	2.45	0.117
	No	23,273 (97.10)	6039 (96.81)	17,234 (97.20)		
Drinking	Yes	3458 (14.37)	873 (13.84)	2585 (14.55)	1.91	0.1467
	No	20,612 (85.63)	5434 (86.16)	15,178 (85.45)		
Folic acid supplementation	Yes	23,606 (96.63)	6148 (96.12)	17,458 (96.81)	6.88	0.009
	No	823 (3.37)	248 (3.88)	575 (3.19)		
Number of pregnancies	Singleton	22,897 (93.73)	6044 (94.50)	16,853 (93.46)	8.69	0.003
	Multiple	1532 (6.27)	352 (5.50)	1180 (6.54)		
Macrosomia history	Yes	28 (0.11)	2 (0.03)	26 (0.14)	5.26	0.022
	No	24,401 (99.89)	6394 (99.97)	18,007 (99.86)		
FBG	Mean ± SD	4.48 ± 0.43	4.83 ± 0.54	4.35 ± 0.0.30	85.88	<0.001
1-h PBG	Mean ± SD	8.04 ± 1.89	10.12 ± 1.55	7.30 ± 1.38	136.05	<0.001
2-h PBG	Mean ± SD	6.90 ± 1.57	8.63 ± 1.52	6.29 ± 1.03	136.98	<0.001

Abbreviations: GDM, gestational diabetes mellitus; BMI, body mass index; FBG, fasting blood glucose; 1-h PBG, 1-h post blood glucose; 2-h PBG, 2-h post blood glucose. Mean ± SD (standard deviation) for continuous variables, and frequency (*n*) and percentage (%) for categorical variables.

**Table 2:** ORs and 95% CI of GDM risk associated with early pregnancy folic acid supplementation.

	Model	OR (95% CI)	*p* Value
Total GDM (*n* = 6396)	1	0.82 (0.70~0.95)	0.009
	2	0.82 (0.71~0.96)	0.012
	3	0.86 (0.74~1.01)	0.063
FBG diagnosed GDM (*n* = 2154)	1	1.00 (0.79~1.25)	0.965
	2	1.05 (0.82~1.34)	0.706
	3	1.13 (0.88~1.46)	0.327
1-h PBG diagnosed GDM (*n* = 3920)	1	0.80 (0.67~0.95)	0.012
	2	0.81 (0.68~0.96)	0.017
	3	0.86 (0.72~1.03)	0.101
2-h PBG diagnosed GDM (*n* = 3857)	1	0.71 (0.60~0.85)	<0.001
	2	0.72 (0.60~0.85)	<0.001
	3	0.75 (0.63~0.90)	0.002

Abbreviations: GDM, gestational diabetes mellitus; FBG, fasting blood glucose; PBG, postprandial blood glucose; OR, odds ratio; CI, confidence interval. Model 1: univariate model; Model 2: not adjust age and pre-pregnancy BMI; Model 3: adjust age, pre-pregnancy BMI, gravidity, parity, smoking history, drinking history, multiple pregnancies, and macrosomia history.

**Table 3:** ORs and 95% CI of GDM risk associated with early pregnancy folic acid supplementation.

Folic Acid Supplementation	N (%)	OR	95% CI	*p*
Average intake, μg/day				
<400	62 (4.75)	1.29	0.72~2.33	0.389
400–800	283 (21.69)	1.32	0.97~1.82	0.081
≥800	960 (73.56)	reference		
Duration, months				
<3	1111 (85.66)	0.93	0.61~1.42	0.742
≥3	186 (14.34)	reference		
Average intake and duration				
<400	62 (4.76)	1.24	0.60~2.57	0.557
400–800, <3	235 (18.04)	1.30	0.74~2.28	0.361
400–800, ≥3	48 (3.68)	1.15	0.51~2.61	0.735
≥800, <3	834 (64.01)	0.95	0.58~1.56	0.847
≥800, ≥3	124 (9.52)	reference		

Abbreviations: OR, odds ratio; CI, confidence interval. Adjust age, pre-pregnancy BMI, gravidity, parity, smoking history, drinking history, multiple pregnancies, and macrosomia history, and gestational week.

## Data Availability

The data presented in this study are available on request from the corresponding author.

## Supplementary Materials

The following supporting information can be downloaded at: https://www.mdpi.com/article/10.3390/nu14194061/s1. Table S1: Demographic characteristics of the study population (*n* = 1305).

## Appendix A

**Table A1:** Sensitivity analysis: potential factors for the impact of folic acid supplementation during early pregnancy on GDM.

	Subgroups		OR (95% CI)	*p* Value
Total GDM	Age (years)	<35	0.89 (0.73~1.09)	0.268
		≥35	0.82 (0.64~1.04)	0.103
	BMI (kg/m^2^)	<25	0.78 (0.66~0.93)	0.006
		≥25	1.07 (0.75~1.52)	0.713
	Pregnancy	Singleton	0.87 (0.74~1.02)	0.084
		Multiple	0.75 (0.38~1.51)	0.422
FBG diagnosed GDM	Age (years)	<35	1.03 (0.75~1.41)	0.856
		≥35	1.31 (0.87~1.98)	0.195
	BMI (kg/m^2^)	<25	1.00 (0.75~1.33)	0.980
		≥25	1.37 (0.85~2.23)	0.196
	Pregnancy	Singleton	1.13 (0.87~1.46)	0.351
		Multiple	1.20 (0.36~4.00)	0.765
1-h PBG diagnosed GDM	Age (years)	<35	0.90 (0.70~1.14)	0.390
		≥35	0.80 (0.61~1.06)	0.118
	BMI (kg/m^2^)	<25	0.78 (0.63~0.95)	0.016
		≥25	1.04 (0.70~1.54)	0.847
	Pregnancy	Singleton	0.86 (0.71~1.04)	0.117
		Multiple	0.82 (0.35~1.91)	0.647
2-h PBG diagnosed GDM	Age (years)	<35	0.84 (0.66~1.12)	0.168
		≥35	0.65 (0.50~0.85)	0.001
	BMI (kg/m^2^)	<25	0.73 (0.60~0.90)	0.002
		≥25	0.72 (0.49~1.05)	0.089
	Pregnancy	Singleton	0.75 (0.62~0.89)	0.001
		Multiple	0.93 (0.40~2.15)	0.866
