# Supplementary material for: Association of Folic Acid Supplementation in Early Pregnancy with Risk of Gestational Diabetes Mellitus: A Longitudinal Study
Source: Nutrients. 2022 Sep 29;14(19):4061. doi: 10.3390/nu14194061 (PMC9571572; doi:10.3390/nu14194061)
Supplement: Supplementary file 1 [file nutrients-14-04061-s001.zip › nutrients-1879566-supplementary.pdf]

Table S1. Demographic characteristics of the study population (n = 1305).

Characteristics		Total	GDM	Non-GDM	χ^2/t	*P*
			N (%)	N (%)		
Age (years)	Means±SD	30.14 ± 4.01	30.73 ± 3.74	29.92 ± 4.08	3.26	0.001
	< 25	85 (6.51)	13 (3.70)	72 (7.55)	14.19	0.003
	25 ~ 30	532 (40.77)	126 (35.90)	406 (42.56)		
	30 ~ 35	492 (37.70)	154 (43.87)	338 (35.43)		
	≥ 35	196 (15.02)	58 (16.52)	138 (14.47)		
Pre-pregnancy BMI (kg/m²)	Means±SD	21.59 ± 3.18	22.41 ± 3.15	21.28 ± 3.14	5.77	<0.001
	< 18.5	216 (16.55)	29 (8.26)	187 (19.60)	38.01	<0.001
	18.5 ~ 23.9	854 (65.44)	232 (66.10)	622 (65.20)		
	24.0 ~ 28	190 (14.56)	77 (21.94)	113 (11.84)		
	≥ 28	45 (3.45)	13 (3.70)	32 (3.35)		
Average gestational weeks	Means±SD	10.98 ± 1.44	10.99 ± 1.40	10.98 ± 1.45	0.07	0.947
Gravidity	1	486 (42.04)	151 (45.21)	335 (40.75)	5.73	0.057
	2	317 (27.42)	98 (29.34)	219 (26.64)		
	≥ 3	353 (30.54)	85 (25.45)	268 (32.60)		
Parity	0	889 (76.64)	261 (77.91)	628 (76.12)	0.43	0.514
	≥ 1	271 (23.36)	74 (22.09)	197 (23.88)		
Smoking	Yes	32 (2.47)	11 (3.15)	21 (2.21)	0.94	0.333
	No	1266 (97.53)	338 (96.85)	928 (97.79)		
Drinking	Yes	202 (15.71)	51 (14.70)	151 (16.08)	0.137	0.545
	No	1084 (84.29)	296 (85.30)	788 (83.92)		
FA supplementation	Yes	1299 (99.54)	349 (99.43)	950 (99.58)	-	0.663
	No	6 (0.46)	2 (0.57)	4 (0.42)		
Number of pregnancy	Singleton	1268 (97.16)	339 (96.58)	929 (97.38)	0.59	0.441
	Multiple	37 (2.84)	12 (3.42)	25 (2.62)		
Macrosomia history	Yes	4 (0.31)	1 (0.28)	3 (0.31)	-	1.000
	No	1301 (99.69)	350 (99.72)	951 (99.69)		
FBG	Mean ± SD	4.50 ± 0.45	4.81 ± 0.66	4.38 ± 0.29	15.40	<0.001
1-h PBG	Mean ± SD	8.20 ± 1.80	10.08 ± 1.67	7.42 ± 1.33	26.50	<0.001
2-h PBG	Mean ± SD	7.03 ± 1.53	8.65 ± 1.62	6.35 ± 1.00	29.71	<0.001

Abbreviations: GDM, gestational diabetes mellitus; BMI, body mass index; FBG, fasting blood glucose; 1-h PBG, 1-hour post blood glucose; 2-h PBG, 2-hour post blood glucose. Mean ± SD (standard deviation) for continuous variables, and frequency (n) and percentage (%) for categorical variables.
